# Association Between Sex Hormone Levels and Clinical Outcomes in Patients With COVID-19 Admitted to Hospital: An Observational, Retrospective, Cohort Study

**DOI:** 10.3389/fimmu.2022.834851

**Published:** 2022-01-27

**Authors:** Anna Beltrame, Pedro Salguero, Emanuela Rossi, Ana Conesa, Lucia Moro, Laura Rachele Bettini, Eleonora Rizzi, Mariella D’Angió, Michela Deiana, Chiara Piubelli, Paola Rebora, Silvia Duranti, Paolo Bonfanti, Ilaria Capua, Sonia Tarazona, Maria Grazia Valsecchi

**Affiliations:** ^1^Department of Infectious, Tropical Diseases and Microbiology Istituto di Ricovero e Cura a Carattere Scientifico (I.R.C.C.S). Sacro Cuore Don Calabria Hospital, Negrar di Valpolicella, Italy; ^2^Department of Applied Statistics, Operations Research and Quality, Universitat Politècnica de València, Valencia, Spain; ^3^Bicocca Center of Bioinformatics, Biostatistics and Bioimaging, School of Medicine and Surgery, Milano-Bicocca University, Milano, Italy; ^4^Institute for Integrative Systems Biology, Spanish National Research Council, Paterna, Spain; ^5^Department of Microbiology and Cell Sciences, University of Florida, Gainesville, FL, United States; ^6^Pediatric Departement and Centro Tettamanti-European Reference Network PaedCan, EuroBloodNet, MetabERN-University of Milano-Bicocca-Fondazione MONZA e BRIANZA per il BAMBINO e la sua MAMMA (MBBM)-Ospedale, San Gerardo, Monza, Italy; ^7^School of Medicine and Surgery, Milano-Bicocca University, Milano, Italy; ^8^Infectious Diseases Unit, Azienda Socio Sanitaria Territoriale (ASST) Monza, San Gerardo Hospital, Monza, Italy; ^9^One Health Center of Excellence, University of Florida, Gainesville, FL, United States

**Keywords:** sex hormones, COVID-19, outcome, ARDS, severity, testosterone, estradiol

## Abstract

Understanding the cause of sex disparities in COVID-19 outcomes is a major challenge. We investigate sex hormone levels and their association with outcomes in COVID-19 patients, stratified by sex and age. This observational, retrospective, cohort study included 138 patients aged 18 years or older with COVID-19, hospitalized in Italy between February 1 and May 30, 2020. The association between sex hormones (testosterone, estradiol, progesterone, dehydroepiandrosterone) and outcomes (ARDS, severe COVID-19, in-hospital mortality) was explored in 120 patients aged 50 years and over. STROBE checklist was followed. The median age was 73.5 years [IQR 61, 82]; 55.8% were male. In older males, testosterone was lower if ARDS and severe COVID-19 were reported than if not (3.6 *vs.* 5.3 nmol/L, p =0.0378 and 3.7 *vs.* 8.5 nmol/L, p =0.0011, respectively). Deceased males had lower testosterone (2.4 *vs.* 4.8 nmol/L, p =0.0536) and higher estradiol than survivors (40 *vs.* 24 pg/mL, p = 0.0006). Testosterone was negatively associated with ARDS (OR 0.849 [95% CI 0.734, 0.982]), severe COVID-19 (OR 0.691 [95% CI 0.546, 0.874]), and in-hospital mortality (OR 0.742 [95% CI 0.566, 0.972]), regardless of potential confounders, though confirmed only in the regression model on males. Higher estradiol was associated with a higher probability of death (OR 1.051 [95% CI 1.018, 1.084]), confirmed in both sex models. In males, higher testosterone seems to be protective against any considered outcome. Higher estradiol was associated with a higher probability of death in both sexes.

## Introduction

Coronavirus disease 19 (COVID-19) emerged in December 2019 as a novel viral pneumonia, caused by severe acute respiratory syndrome coronavirus-2 (SARS-CoV-2) (1). Epidemiological studies have shown that clinical manifestations of SARS-CoV-2 include asymptomatic infection, bilateral pneumoniae, acute respiratory distress syndrome (ARDS), and death (1). Studies worldwide have shown that sex is an important risk factor in COVID-19 outcomes (1–4). Klein and colleagues reported a significant male-female difference in COVID-19 cases, hospitalizations, and deaths (5). Men were more likely to require intensive care than women, and also had a higher mortality rate (6). Sex disaggregated data from 190 countries confirmed a greater mortality rate in men, especially if over 50 years of age (7).

Social-behavioral (gender) and biological (sex) factors have been considered possible causes of this disparity (3, 8). Women develop a stronger innate and adaptive immune response to infections, which is associated with an increased risk of developing autoimmune diseases (9). The immune response to viral infections varies with sex hormone fluctuations, as they control both cellular and humoral components (10). For this reason, sex hormones have been considered essential in many infectious disease outcomes (11–13). Low testosterone and high estradiol levels have been already shown to be related with a worse outcome in males with severe sepsis (12).

Our study hypothesized that sex hormone levels could be associated with the clinical outcomes of COVID-19 patients. We analyzed four sex hormones in hospitalized COVID-19 patients to determine whether their levels were associated with clinical outcomes.

## Material and Methods

The Strengthening the Reporting of Observational Studies in Epidemiology (STROBE) guidelines were used (14).

### Study Design and Setting

This is an observational, retrospective, cohort study conducted at the Department of Infectious, Tropical Diseases and Microbiology, IRCCS Sacro Cuore Don Calabria Hospital of Negrar di Valpolicella, Italy, and at the San Gerardo Hospital of Monza, Italy. The study was conducted within the Circular Health Initiative promoted by the University of Florida One Health Center (https://onehealth.ifas.ufl.edu/activities/circular-health-program/circular-health/sex-differences/).

### Participants and Data

Analyzed samples were taken from patients (aged 18 years or older) hospitalized at the IRCCS Sacro Cuore Don Calabria Hospital (77) and at the San Gerardo Hospital, ASST Monza (61), with a confirmed SARS-CoV-2 infection. A stored serum or plasma sample from these patients was collected upon arrival (or during the first two days after admission) through the first wave of the COVID-19 pandemic (between February 1 and May 30, 2020). Patients who did not consent to the anonymous storage of serum/plasma samples for research purposes were not considered. Patients with an insufficient blood sample for the sex hormone analysis or with missing clinical data also were excluded.

Data up to discharge from hospital or death during hospitalization were gathered from health records in a predefined electronic database. The IRCCS Sacro Cuore Don Calabria Hospital adopted the case report form developed by the International Severe Acute Respiratory and Emerging Infection Consortium (ISARIC) and WHO for use in outbreak investigations (15); the San Gerardo Hospital developed an *ad-hoc* platform on the basis of WHO COVID-19 (COVID-STORM, ClinicalTrials.gov:NCT04424992).

The variables considered in this study were: demographic, clinical characteristics, and symptoms at entry; date of symptoms onset; vital signs upon emergency room admission; laboratory data from the first two days of hospital admission; chest X-ray and computed tomography (CT) results at entry; illness severity; development of pulmonary complication (ARDS); any supplemental oxygen or form of respiratory support; intensive care unit (ICU) admission with length.

The outcomes considered were: ARDS; severe COVID-19; in-hospital mortality. A full description of the definitions is in the **Supplementary Material**.

Potential confounders included age, the number of comorbidities, and viral pneumonia (defined as the presence of a new or progressive infiltrate on a chest X-ray or CT) at baseline, due to their known association with a worse outcome (16).

The levels of four sex hormones (estradiol, testosterone, progesterone, dehydroepiandrosterone) were tested on the stored blood samples according with the approach described in the **Supplementary Material**.

### Ethical Consideration

This observational study required no change to the clinical management of participating patients and did not prevent patients from participating in other research. Ethical approval for data collection was obtained in accordance with local regulations at each participating site. The study protocol received ethical approval and consent from the competent Ethics Committee (*Comitato Etico per la sperimentazione Clinica delle Province di Verona e Rovigo*) on September 1^st^, 2020 (protocol #46555).

### Statistical Analysis

The sex hormone description included the initial cohort of 138 patients, while a subsequent analysis was done on a sub-cohort of patients aged 50 years and over (120 patients).

Patients’ characteristics and sex hormone levels were described by frequencies and percentages for categorical variables, or median and interquartile range [IQR, first - third quartile] for continuous variables.

Differences between the sexes were tested by applying the chi-squared or Fisher’s test when appropriate, and the Wilcoxon rank-sum test for categorical and continuous variables, respectively. Adjusted false discovery rate (FDR) p-values were used for laboratory data.

Logistic regression models were used to estimate the effect of three sex hormones (testosterone, estradiol, and progesterone) on the following outcomes: i) the development of ARDS, ii) severe COVID-19 (ordinary scale 3 = no, 4-7 = yes), and iii) in-hospital mortality. The three models were performed on both the entire population and each sex subgroup. Models were adjusted for sex (in the full model), age, number of comorbidities, and diagnosis of viral pneumonia at entry. Odds Ratio (OR), 95% confidence interval and p-values were reported. P-values were considered statistically significant when lower than 0.05. Statistical analyses were done by SASv9.4 and R.

## Results

### Description of the Overall Cohort

**Figure 1:**
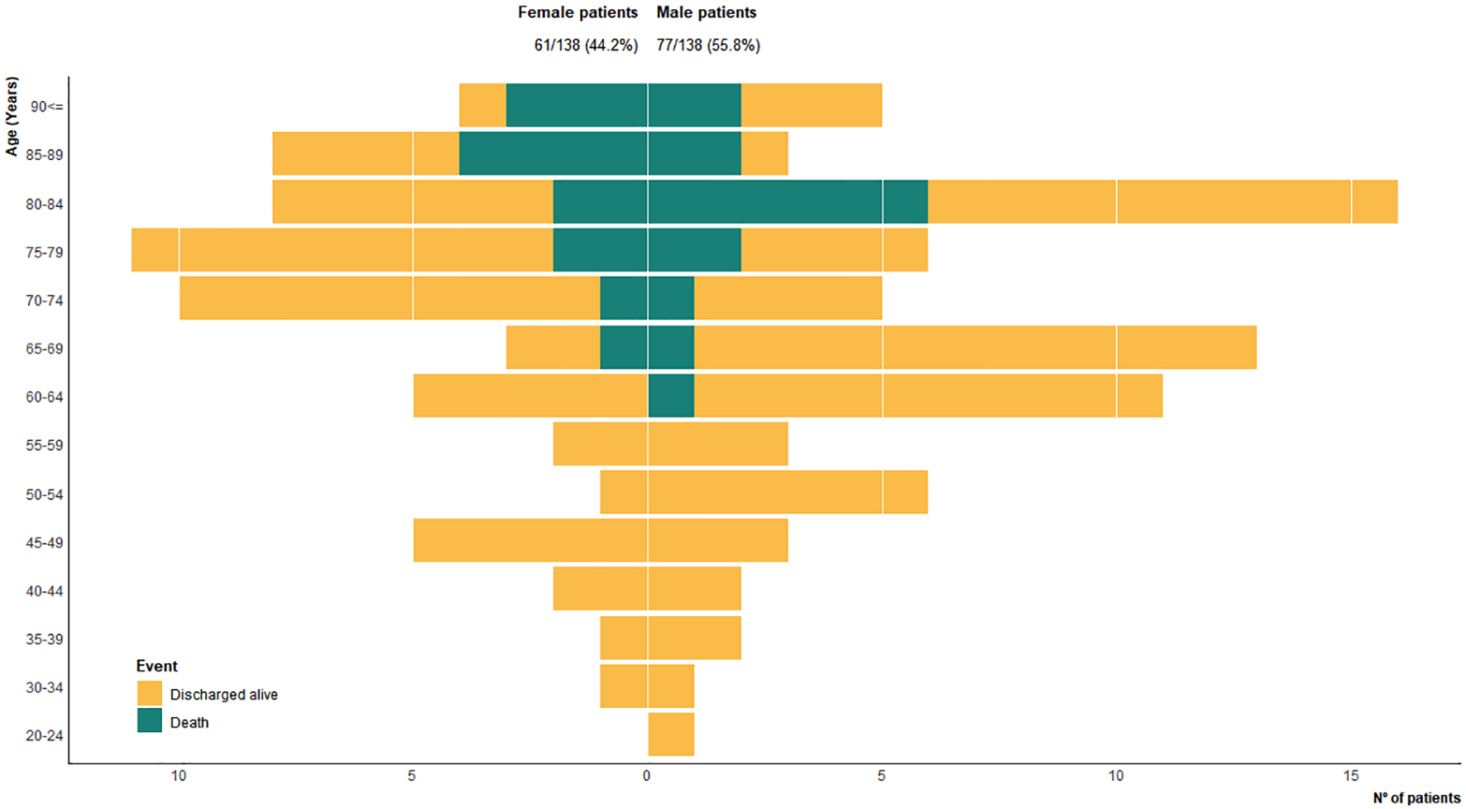
Sex distribution for 138 hospitalized COVID-19 patients in 5-year age groups, according to outcome: discharged; alive; dead. Bar fills are outcomes.

The study population included 138 patients with a confirmed SARS-CoV-2 infection. The median age was 73.5 years [IQR 61, 82]; 61 patients (44.2%) were female (median age 75 years) and 77 (55.8%) were male (median age 68 years). Only 18 patients were under the age of 50 (nine for each sex) (**Figure 1**).

The median testosterone level in males both younger and older than 50 years was below the lower limit of the normal range of each age group (4·9 and 4·4 nmol/L, respectively) (**Table 1**). Testosterone deficiency (**Supplementary Material**) was evident in six (75%) males aged 18 to 49 years (n=8), and 58 (86.6%) in those older (n=67). No other variation was evident for the remaining sex hormones. No departure of the four sex hormones from the normal range was described in the female group.

**Table 1:** Median sex hormone levels of 138 patients, stratified by sex and age.

	Female N=61				Male N=77			
	21-49 years old (n = 9)	Normal range	≥ 50 years old (n = 52)	Normal range	21-49 years old (n = 9)	Normal range	≥ 50 years old (n = 68)	Normal range
**Sex hormone levels, median (IQR)**								
Testosterone, nmol/L	0.7 (0.6-0.8)	0.48-1.85	0.9 (0.7-1.4)	0.43-1.24	4.9 (4.8-10.2)	8.33-30.19	4.4 (1.5-7.2)	7.66-24.82
Estradiol, pg/mL	68 (39-87)	21-649	20 (14-35)	<10-28	29 (22-35)	11-44	28 (21-38.5)	11-44
Progesterone, ng/mL	0.2 (0.1-0.4)	<0.1-15.9	0.1 (0.1-0.3)	<0.1-0.2	0.1 (0.1-0.3)	<0.1-0.2	0.1 (0.1-0.2)	<0.1-0.2
DHEA, microg/dL	107 (66.6-121.3)	56.2-511.7	66 (40.8-102.5)^*^	29.7-282.9	184.8 (78.8-196.7)^#^	136.2-591.9	69.2 (43.8-108.8)^^^	48.6-447.6

*N=51; #N=8; ^N=66.

Since the development of ARDS in the smaller subset of patients younger than 50 occurred in only four males and none died from this age group (**Figure 1**), the following analyses were narrowed to patients aged 50 years or older.

### Results on Patients Aged 50 Years and Older

One hundred and twenty patients were aged 50 years and older, 52 (43.3%) females (median age 77, [IQR 71.5, 84]) and 68 (56.7%) males (median age 70.5, [IQR 63.5, 82]). Age groups, the number of comorbidities, and symptoms are summarized in **Table 2**. The percentage of patients older than 70 years was higher in females than males (76.9 *vs*. 50%, p=0.00026). Ninety-two (78%) patients had viral pneumoniae at baseline (**Table 2**), with a higher incidence in males compared to females (86.8 *vs*. 69.2%, p-value =0.0005).

**Table 2:** Main characteristics at baseline in 120 patients 50 years and over, stratified by sex.

Patients characteristic at baseline, n (%)	Overall n = 120	Sex		p-value
		Female n = 52	Male n = 68	
**Age groups, years**				
50-70	46 (38.3)	12 (23.1)	34 (50.0)	0.0026
>70	74 (61.7)	40 (76.9)	34 (50.0)	
**Number of comorbidities**				
0	18 (15.0)	8 (15.4)	10 (14.7)	0.5628
1	24 (20.0)	13 (25.0)	11 (16.2)	
2-3	57 (47.5)	24 (46.1)	33 (48.5)	
>3	21 (17.5)	7 (13.5)	14 (20.6)	
**Number of symptoms**				
0	17 (14.1)	9 (17.3)	8 (11.8)	0.8214
1	23 (19.2)	11 (21.1)	12 (17.6)	
2-3	54 (45.0)	21 (40.4)	33 (48.5)	
3-5	20 (16.7)	9 (17.3)	11 (16.2)	
>5	6 (5.0)	2 (3.9)	4 (5.9)	
**Pneumonia**				
Yes	95 (79.2)	36 (69.2)	59 (86.8)	0.0005
No	25 (20.8)	16 (30.8)	9 (13.2)	

Details on individual comorbidities, symptoms, and vital signs at baseline are summarized in the **Supplementary Material**, as well as laboratory data, both stratified by sex.

**Table 3** presents the gravity of COVID-19, including the type of respiratory treatment, ICU admission, and clinical outcomes (ARDS, severe COVID-19, and in-hospital mortality) for both the overall population and stratified sex groups. Ninety-six (80%) patients received some form of supplemental oxygen during hospitalization, with no significant difference by sex. Eight (11·8%) males required invasive ventilation, while such treatment was not necessary for any female. ICU admission was required for 12 patients, all male (17.7%), who had a median hospital stay of 8·5 days [IQR 5.75, 13.5]. As for out outcome indicators, 53 (44.5%) patients developed ARDS with a higher incidence in males compared to females (52.2 *vs*. 34.6%, p-value =0.0550). In particular, males had a significantly more severe form of ARDS than females (28.4 *vs*. 6%, p-value =0.0107). Ninety-seven (80.8%) patients had severe COVID-19 and the in-hospital mortality rate was 23·3%. For both outcomes, no statistically significant difference between the sexes was found (**Table 3**).

**Table 3:** Type of respiratory support (oxygen therapy and invasive ventilation), ICU admission, and clinical outcomes (ARDS, severe COVID-19, and in-hospital mortality) of patients 50 years and over, stratified by sex (if data are known on a subset of patients, the number is indicated in parenthesis after the variable name).

Outcome, n (%)	Overall n=120 (%)	Sex		P-value
		Female n=52 (%)	Male n=68 (%)	
**Type of treatment**				
**Any oxygen therapy**				
NO	24 (20.0)	14 (26.9)	10 (14.7)	0.0973
YES	96 (80.0)	38 (73.1)	58 (85.3)	
**Invasive ventilation**				
NO	112 (93.3)	52 (100.0)	60 (88.2)	0.0097
YES	8 (6.7)	0 (0.0)	8 (11.8)	
**ICU admission**				
NO	108 (90)	52 (100.0)	56 (82.4)	0.0023
YES	12* (10)	0 (0.0)	12 (17.7)	
**ARDS (119/120)**				
NO	66 (55.5)	34 (65.4)	32 (47.8)	0.0550
YES	53 (44.5)	18 (34.6)	35 (52.2)	
**ARDS severity (117/120)**				
No ARDS	66 (56.4)	34 (68.0)	32 (47.8)	0.0107
Mild ARDS	20 (17.1)	10 (20.0)	10 (14.9)	
Moderate ARDS	9 (7.7)	3 (6.0)	6 (9.0)	
Severe ARDS	22 (18.8)	3 (6.0)	19 (28.4)	
**Severe COVID-19**				
NO	23 (19.2)	11 (21.1)	12 (17.6)	0.6287
YES	97 (80.8)	41 (78.9)	56 (82.4)	
**In-hospital mortality**				
NO	92 (76.7)	39 (75.0)	53 (77.9)	0.7058
YES	28 (23.3)	13 (25.0)	15 (22.1)	

*median ICU staying duration is equal to 8.5 days [IQR 5.75, 13.5].

### Association Between Sex Hormones and Clinical Outcomes, Stratified by Sex

Sex hormone levels according to outcome (ARDS, severe COVID-19, in-hospital mortality), in females and males separately, are displayed in **Table 4**.

**Table 4:** Sex hormone levels in patients aged 50 years and over, stratified by sex and outcome.

Sex hormones median (IQR)	Female* (n=52)				Male# (n=68)				
	Testosterone nmol/L	Estradiol pg/mL	Progesterone ng/mL	DHEA microg/L	Testosterone nmol/L		Estradiol pg/mL	Progesterone ng/mL	DHEA microg/L
**Normality range**	**0.43-1.24**	**<10-28**	**<0.1-0.2**	**29.7-282.9**	**7.7-24.8**		**11-44**	**<0.1-0.2**	**48.6-447.6**
**ARDS (119/120)**									
NO	0.9 (0.7-1.6)	26 (15-41)	0.1 (0.1-0.2)	66.0 (44.8-106.9)	5.3 (1.8-11.1)		24 (19.5-33.0)	0.1 (0.1-0.2)	69.3 (32.5-96.2)
YES	0.9 (0.6-1.1)	18.5 (10-21)	0.2 (0.1-0.3)	61.2 (30.3-79.1)	3.6 (1.3-6.2)		31 (23.0-40.0)	0.1 (0.1-0.2)	69.4 (50.7-118.3)
p-value	0.2182	0.0459	0.2038	0.2740	0.0378		0.1270	0.9008	1
**Severe COVID-19**									
NO	0.7 (0.6-4.7)	18 (10-31)	0.1 (0.1-0.2)	86.5 (52.9-105.4)	8.5 (5.9-12.4)		23.5 (16.5-27.5)	0.2 (0.1-0.3)	67.1 (23.1-171.5)
YES	0.9 (0.7-1.3)	20 (14-35)	0.2 (0.1-0.3)	63.7 (38.2-103.25)	3.7 (1.3-6.3)		30.0 (22.0-41.5)	0.1 (0.1-0.2)	69.4 (50.7-100.1)
p-value	0.9911	0.4187	0.1092	0.4707	0.0011		0.3146	0.0176	0.8905
**In-hospital mortality**									
NO	0.8 (0.6-1.3)	20 (15-30)	0.1 (0.1-0.2)	69.0 (40.8-105.4)	4.8 (1.5-7.9)		24.0 (20.0-33.0)	0.1 (0.1-0.2)	76.6 (42.3-117.2)
YES	1.2 (1.0-1.7)	36 (11-64)	0.2 (0.1-0.4)	61.4 (48.8-96.4)	2.4 (1.3-4.6)		40.0 (30.0-52.0)	0.2 (0.1-0.2)	62.6 (32.5-91.9)
p-value	0.1418	0.4520	0.4231	0.7376	0.0536		0.0006	0.1618	0.6029

*Female: 18/52 ARDS; 41/52 severe COVID-19, 13/52 in hospital mortality.

^#^Male: 35/68 ARDS; 56/68 severe COVID-19, 15/68 in hospital mortality.

No statistically significant difference in testosterone levels was evident in the female population that developed ARDS, severe COVID-19, or those that died compared to females with more favorable outcomes.

**Figure 2:**
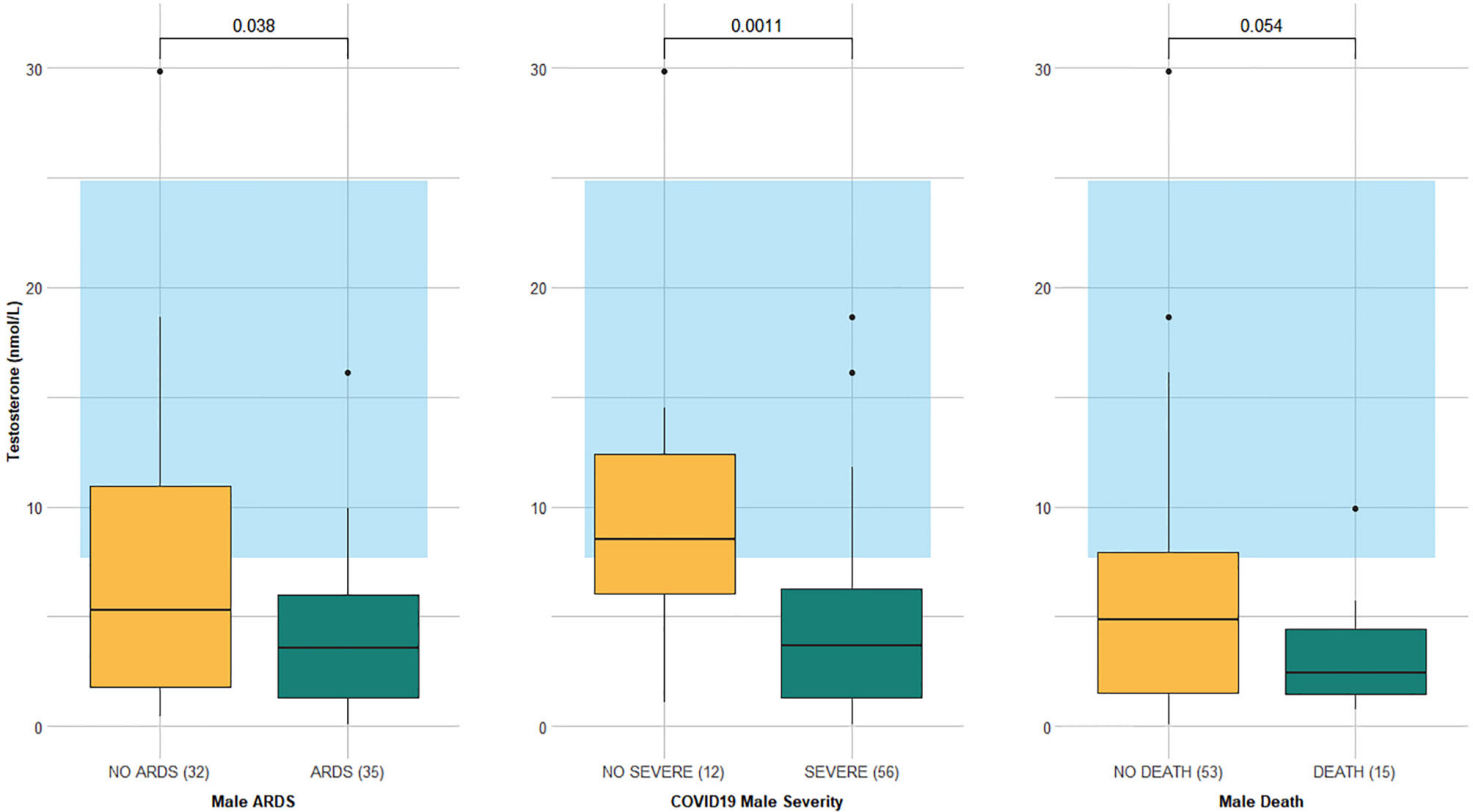
Testosterone levels according to outcomes (ARDS, severe COVID-19, and in-hospital mortality) in 68 males aged 50 years and older. Data are expressed as box plot with median (cross line) and interquartile ranges; whiskers are the minimum and maximum values. The laboratory-assessed hormone reference ranges are indicated in light blue. Circles represent the outliers.

The testosterone level was significantly lower in males who developed ARDS and severe COVID-19 than in those who did not (3.6 *vs*. 5.3 nmol/L, p-value =0.0378 and 3.7 *vs*. 8.5 nmol/L, p-value =0.0011, respectively), while it was borderline significant in the deceased versus survivors (2.4 *vs*. 4.8 nmol/L, p-value =0.0536) (**Table 4** and **Figure 2**).

The estradiol level was lower in females with ARDS than in those without it (18.5 *vs*. 26 pg/mL, p-value =0.0459), but similar in females who either developed or did not develop severe COVID-19. There was no statistically significant difference in the estradiol level of deceased versus surviving females, although in the former it was higher (36 *vs*. 20 mmol/L, p-value =0.4520), with a median value above the upper limit of the normal range. In males, the estradiol level was similar in patients who suffered both ARDS and severe COVID-19 compared to those who did not. In contrast, the deceased had a significantly higher estradiol level compared to survivors (40 *vs*. 24 pg/mL, p-value = 0.0006).

No statistically significant difference in progesterone level was evident in the females for any of the clinical outcomes considered. Differently, males who developed severe COVID-19 had a lower progesterone level compared to mild forms of the disease (0.1 *vs*. 0.2 pg/mL, p-value = 0.0176).

The DHEA level did not change in either sex for any clinical outcome and was thus not considered in the regression models.

**Table 5** shows the results of the multivariable logistic regression models considering each outcome variable, for the entire population of patients aged 50 and over, and on both male and female subgroups.

**Table 5:** Logistic models on ARDS, severe COVID-19 and in-hospital mortality, overall and in the subset of male and female patients.

Overall Model	ARDS		SEVERE COVID		IN-HOSPITAL MORTALITY	
	ODDS RATIO (95%CI)	P-value	ODDS RATIO (95%CI)	P-value	ODDS RATIO (95%CI)	P-value
	N=117/120		N=118/120		N=118/120	
**Sex: Male *vs* Female**	3.151 (1.050-9.459)	0.0407	3.068 (0.516-18.226)	0.2176	1.823 (0.488-6.806)	0.3719
**Testosterone**	0.849 (0.734-0.982)	0.0274	0.691 (0.546-0.874)	0.0021	0.742 (0.566-0.972)	0.0300
**Estradiol**	0.979 (0.957-1.002)	0.0694	1.079 (1.000-1.164)	0.0504	1.051 (1.018-1.084)	0.0020
**Progesterone**	1.636 (0.462-5.793)	0.4451	1.055 (0.580-1.920)	0.8609	0.136 (0.005-3.527)	0.2298
**Age**	1.022 (0.979-1.068)	0.3194	1.028 (0.970-1.089)	0.3525	1.141 (1.065-1.223)	0.0002
**N. of comorbidities**	1.188 (0.868-1.626)	0.2830	1.286 (0.858-1.926)	0.2234	1.000 (0.690-1.450)	0.9994
**Pneumonia at entry**	13.013 (3.348-50.575)	0.0002	7.758 (1.927-31.236)	0.0039	1.700 (0.409-7.057)	0.4653
**Model on males**	**N=67/68**		**N=67/68**		**N=67/68**	
**Testosterone**	0.874 (0.763-1.000)	0.0501	0.617 (0.347-0.872)	0.0062	0.705 (0.519-0.959)	0.0258
**Estradiol**	0.999 (0.967-1.033)	0.9610	1.112 (0.965-1.281)	0.1424	1.068 (1.010-1.129)	0.0219
**Progesterone**	1.107 (0.323-3.801)	0.8713	0.382 (0.100-1.462)	0.1599	<0.001 (<0.001-1.395)	0.0603
**Age**	1.000 (0.948-1.054)	0.9920	1.017 (0.929-1.113)	0.7181	1.145 (1.044-1.256)	0.0042
**N. of comorbidities**	1.541 (1.018-2.334)	0.0411	2.153 (1.006-4.607)	0.0482	1.196 (0.713-2.006)	0.4972
**Pneumonia at entry**			24.921 (1.684-368.755)	0.0193	1.001 (0.061-16.309)	0.9994
**Model on females**	**N=51/52**		**N=51/52**		**N=51/52**	
**Testosterone**	0.445 (0.067 - 2.960)	0.4020	0.524 (0.182 - 1.506)	0.2300	0.578 (0.121 - 2.759)	0.4914
**Estradiol**	0.962 (0.906 - 1.022)	0.2090	1.076 (0.959 - 1.207)	0.2101	1.058 (1.007 - 1.111)	0.0245
**Progesterone**	2.724 (0.223 - 33.322)	0.4329	1.245 (0.341 - 4.544)	0.7405	0.144 (0.026 - 0.791)	0.0258
**Age**	1.034 (0.962 - 1.112)	0.3593	1.030 (0.948 - 1.120)	0.4832	1.169 (1.030 - 1.328)	0.0158
**N. of comorbidities**	0.812 (0.481 - 1.372)	0.4374	1.050 (0.571 - 1.932)	0.8747	0.976 (0.514 - 1.854)	0.9416
**Pneumonia at entry**	8.808 (1.597 - 48.581)	0.0125	4.313 (0.775 - 24.004)	0.0951	2.000 (0.319 - 12.556)	0.4594

After adjusting for possible confounders, higher levels of testosterone were protective against each of the three unfavorable outcomes in the entire population. For each nmol/L increment in testosterone level, the odds of a worse outcome significantly decreased by 15% for ARDS, 31% for severe COVID-19, and 26% for in-hospital mortality. Higher estradiol was associated with a slightly higher risk of death during hospitalization (OR 1.051 [95% CI 1.018, 1.084]), while this hormone had no significant impact on the development of severe COVID-19 or ARDS. Progesterone did not have a significant prognostic effect on the clinical outcomes considered. Males had a higher risk of developing an unfavorable outcome, but this was significant only for ARDS (OR 3.151 [95% CI 1.050, 9.459]). Older age was associated with an increased risk of death (OR 1.141 [95% CI 1.065, 1.223]), while the presence of viral pneumonia at baseline greatly increased the risk of developing ARDS (OR 13.013 [95% CI 3.348, 50.575]) or severe COVID-19 (OR 7.758 [95% CI 1.927, 31.236]).

Analyzing only the male subgroup, the protective effect of testosterone was notable for all outcomes, while estradiol played a role similar to that found in the entire cohort (**Table 5**). In the subset of females, the logistic model shows an association between higher levels of estradiol and a higher risk of death during hospitalization (OR 1.058 [95% CI 1.007, 1.111]). However, for each ng/mL increment in progesterone, the odds of dying significantly decreased by 86% (OR 0.144 [95% CI 0.026, 0.791]).

## Discussion

A significant male-female difference in the severity of COVID-19 cases has been reported in medical literature (1–6). The question arises whether these clinical differences correspond to diverse sex hormone levels.

Firstly, we evaluated the sex hormone levels of hospitalized COVID-19 patients, differentiated by age and sex. Testosterone levels were severely impaired in all males regardless of age and contrasted the other three sex hormones, each being within the normal range. In particular, 75% of males under the age of 50 and 87% of those over 50 had testosterone deficiency. On the other hand, no changes were found for all sex hormone levels from the reference values in the female group.

Low testosterone levels have been reported during surgical stress and other critical illnesses, possibly to reduce energy consumption for survival (17). Furthermore, a progressive decline in testosterone levels occurs during the aging process, due to primary (testicular origin) and/or secondary hypogonadism (hypothalamic-pituitary origin) (18). Reduced testosterone levels have been also observed in patients with cardiovascular disease, obesity, type II diabetes, and chronic obstructive pulmonary disease (19, 20). Given the high number of comorbidities reported by the males aged 50 years and older (85% had ≥ one) and a median age of 70.5 years in this subgroup, it is not surprising that their testosterone levels were very low.

Moreover, the virus per se can also alter testosterone levels. When SARS-CoV-2 replicates in the reproductive organs of animals, it causes massive dysregulation of sex hormones and induces an elevated transcription of CYP19A1 aromatase in the lungs, the enzyme responsible for converting testosterone to estradiol (21). This event occurs particularly in male hamsters, severely depleting testosterone and highly elevating estradiol levels. However, in female hamsters, it reduces estradiol levels only. CYP19A1 aromatase is more elevated in males than females, either animals or humans. However, in male animals, this shift occurs specifically with SARS-CoV-2. In fact, injecting other viruses in male mice has been shown to cause a reduction in testosterone levels, with no alteration in estradiol (21).

Lower testosterone in COVID-19 patients than controls has also been reported in small observational studies (22, 23). Kadihasanoglu and colleagues found a lower median testosterone level in 89 COVID-19 patients (185.5 ng/dL) compared to the level found in 30 patients with other respiratory diseases (288.7 ng/dL) and to that 143 age-matched healthy controls (332 ng/dL) (22). In particular, the proportion of patients with a testosterone deficiency was 74.2, 53.3, and 37.8%, respectively (p-value<0.0001) (22). In 50 COVID-19 patients (39 males and 11 females) requiring ICU admission, plasma testosterone levels were strongly reduced in males compared to healthy controls and non-COVID-19 patients with coronary heart disease (23). This difference was not evident in the female group. Differently from healthy controls and patients with coronary heart disease, plasma estradiol levels were significantly increased in both COVID-19 males and females (23).

Several studies have shown that age and comorbidities are important risk factors for death in patients with COVID-19 (16). However, few studies investigating the relationship between sex hormones and COVID-19 outcomes are available (24–29). Therefore, the second goal of our study was to explore whether sex hormone levels were associated with COVID-19 outcomes. The analysis was restricted to the population aged 50 years and older, as younger patients rarely had respiratory complications, and none died. In our older cohort of 120 patients, we found no significant difference in the death rate between sexes, likely due to limitations in numbers of patients and events. However, ARDS, which often requires ICU admission, occurred more in males, which is consistent with various studies (1–7). In particular, testosterone levels were significantly lower in the male population that developed ARDS and severe COVID-19 than in those who did not.

The role of sex hormones in COVID-19 outcomes remains controversial, as it has only been analyzed in small studies including patients with differing median ages (24–26).

Rastrelli and colleagues studied the association between testosterone levels and outcomes in 31 males with COVID-19 pneumoniae admitted to the Respiratory ICU (24). A stepwise decrease in total testosterone was evident according to COVID-19 severity: 8.8 nmol/L in 21 patients who clinically improved; 5 nmol/L in six patients remaining on non-invasive ventilation therapy; 1 nmol/L in four patients requiring intubation, or who died. In a case-control study, the median testosterone level was significantly lower in the COVID-19 cases (1.4 *vs*. 3.5 ng/mL, p-value=0.005), yet there was no difference in the median estradiol level between 24 patients with severe COVID-19 pneumoniae and 24 controls (30 *vs*. 28 pg/mL, p-value=0.959). Still, the small population size inhibited the discovery of any association between testosterone and death (25). In a retrospective study including 221 hospitalized patients with SARS-CoV-2 infection, the testosterone levels were 346 ng/dL in 46 asymptomatic patients, 318 ng/dL in 129 patients hospitalized in internal medicine, and 241 ng/dL in 46 ICU patients (26). As the testosterone level decreased, the probability of being admitted to the ICU or dying increased significantly (p-value=0.001 and p-value=0.002, respectively). No differences were found for estradiol levels (26).

Our overall multivariable logistic regression analysis estimated that for each nmol/L increment in testosterone level, the odds of developing ARDS, severe COVID-19, and in-hospital mortality decreased by 15, 31, and 26%, respectively, after adjustment for main confounders. The same analysis on the subset of males obtained similar results (13, 38, and 29%, respectively) confirming the protective role of testosterone. Differently, for each pg/mL increase of estradiol, the odds of dying during hospitalization increased by five percent, without impacting the other two outcomes. This last result was confirmed in both sex models.

Only three observational studies have suggested that testosterone levels, and to a lesser extent, estradiol levels, are associated with COVID-19 outcomes (27–29). The small retrospective study of Salsiccia and colleagues investigated total testosterone levels of 29 hospitalized COVID-19 men, nine requiring invasive oxygenation treatment and 20 requiring no oxygenation therapy (27). After adjusting for the Age-adjusted Charlson Comorbidity index, hypertension, dyslipidemia, and smoking status, a higher total testosterone level was independently associated with lower odds of invasive oxygenation, corresponding to a better outcome (OR 0.43 [95% CI, 0.23-0.85]) (27).

In a larger prospective cohort study investigating 358 COVID-19 males, the testosterone level was statistically significantly lower in those admitted to the ICU compared to those who were not (64 *vs*. 286 ng/dl, p-value < 0.001), as well as the deceased compared to survivors (82.9 *vs*. 166 ng/dl; p-value < 0.001) (28). The multivariate binary logistic regression analysis found an association between testosterone and ICU admission (OR 0.985 [95% CI, 0.985, 0.993]), as well as testosterone and mortality (OR 0.989 [95% CI, 0.989, 0.998]) (28). In our study, to reduce the risk of bias, ICU admission was not considered as an outcome. In fact, during the first wave of COVID-19, the limited availability of ICU beds meant that younger patients with greater chances of survival may have been admitted over others, caused by the unexpected number of severe patients arriving in a short period of time.

In the prospective cohort study of Dhindsa and colleagues, which included 152 COVID-19 patients, the median testosterone level among men with severe COVID-19 was lower compared to those with milder disease (53 *vs*. 151 ng/dL, p-value=0.01), whereas no estradiol difference was found in the same male groups (29). Testosterone levels in males were inversely associated with severe COVID-19 (OR 0.11 [95% CI, 0.02, 0.059]), ICU admission (OR 0.15 [95% CI, 0.04, 0.57]), ventilator use (OR 0.29 [95% CI, 0.11, 0.81]), and death (OR 0.41 [95% CI, 0.16, 1.03]), independently of other risk factors. Otherwise, estradiol levels were no different in these males. In the female population, no alteration of any sex hormone level was found and no association between hormone levels and outcomes was observed (29). In contrast with these results, our multivariable analysis showed that a higher estradiol level was associated with a higher probability of death in both the male (OR 1.068 [95% CI, 1.010, 1.129]) and female models (OR 1.058 [95% CI, 1.007, 1.111]).

However, the results of our study must take some limitations into account. First and foremost is the small size of the study population and the relatively small number of deaths, especially in the female population 50 years and over. Second, the sex hormone analysis was based on a single measurement upon hospital admission. Third, possible hormone treatment and other factors, such as stress, that may have influenced the hypothalamic-pituitary-gonadal axis were not recorded or measurable. Finally, the retrospective nature of the study and the availability of a limited number of potential confounders consistently collected on all patients due to the chaotic nature of the first pandemic wave.

In conclusion, our study shows that in males aged 50 years and over, higher testosterone protects against unfavorable outcomes, whereas higher estradiol is associated with a higher probability of in-hospital death, regardless of sex. Our study reinforces the recommendation that pre-admission screening for low testosterone could aid the identification of men at high risk of severe COVID-19 as has been already proposed by and colleagues (30). It remains unknown whether sex hormone levels are simple markers or have a causal role in changing outcomes. To confirm the prognostic role of these hormones in clinical practice, larger prospective studies must focus on the level of testosterone, estradiol, and progesterone in hospitalized COVID-19 patients, stratified by sex and age, and according to well defined clinical outcomes. Furthermore, studies on using hormone replacement or suppressive therapy for the treatment of serious COVID-19 may be also planned once stronger association evidence is available. Finally, although our study investigated patients aged 50 years and over, the fact that low testosterone levels were also observed in the younger population suggests that low hormone levels may also play a role in their COVID-19 outcomes. As teenagers and young adults are not yet vaccinated in all countries, or in certain places where the population at large remains at risk, it may also be important to launch studies on hormonal levels in the young.

## Data Availability Statement

The raw data supporting the conclusions of this article will be made available by the authors, without undue reservation.

## Ethics Statement

The studies involving human participants were reviewed and approved by Comitato Etico per la sperimentazione Clinica delle Province di Verona e Rovigo) on September 1st, 2020 (protocol #46555). The patients/participants provided their written informed consent to participate in this study.

## Author Contributions

AB, LM, and IC conceived the work. AB, LM, MV, and AC contributed to the protocol development, study design, and concepts. AB, LM, SD, and PB collected the data. LB, MD’A, ElR, and MD collected and stored the blood samples. ElR and MD analysed sex hormone levels in the blood samples and prepared the dataset. PS integrated data from hospitals, curated datasets, and created paper tables and figures. PS and EmR performed the statistical analysis. ST, MV, PR, and AC supervised statistical methods and data analysis. AB, MV, EmR, PS, and AC interpreted the data. AB searched the literature and wrote the manuscript draft. MV, PS, EmR, AC, LM, and CP contributed to manuscript writing. They revised and edited the manuscript critically. All authors confirm that they had full access to all the data and accept responsibility for the publication submission. All authors contributed to the article and approved the submitted version.

## Funding

The research was funded by Italian Ministry of Health “Fondi Ricerca Corrente, Project L1P5” for IRCCS Sacro Cuore Don Calabria Hospital. The funding source had no role in the collection, analysis, or interpretation of data, the study design, or the writing of the paper.

## Conflict of Interest

The authors declare that the research was conducted in the absence of any commercial or financial relationships that could be construed as a potential conflict of interest.

## Publisher’s Note

All claims expressed in this article are solely those of the authors and do not necessarily represent those of their affiliated organizations, or those of the publisher, the editors and the reviewers. Any product that may be evaluated in this article, or claim that may be made by its manufacturer, is not guaranteed or endorsed by the publisher.
