# Supplementary material for: Association Between Sex Hormone Levels and Clinical Outcomes in Patients With COVID-19 Admitted to Hospital: An Observational, Retrospective, Cohort Study
Source: Front Immunol. 2022 Jan 27;13:834851. doi: 10.3389/fimmu.2022.834851 (PMC8829540; doi:10.3389/fimmu.2022.834851)
Supplement: Supplementary file 1 [file DataSheet_1.docx]

**Supplementary material**

**1.0 Definitions**

SARS-CoV-2 infection was defined if SARS-CoV-2 virus was detected in the nasopharyngeal swab specimen through real-time polymerase chain reaction (rt-PCR) method, performed in accordance with World Health Organization (WHO) protocol.

Because of the expected sex hormone status, two age groups representing probable reproductive and non-reproductive (or less reproductive) groups were defined: 18-49 years old and aged 50 years and over.

ARDS was defined according to the Berlin criteria^1^, in which illness severity is described by the level of hypoxemia measured:

- Mild ARDS: PaO_2_/FiO_2_ >200 mmHg, but ≤300 mmHg, on ventilator settings that include positive end expiratory pressure (PEEP) or continuous positive airway pressure (CPAP) ≥5 cm H2O.
- Moderate ARDS: PaO_2_/FiO_2_ >100 mmHg, but ≤200 mmHg, on ventilator settings that include PEEP ≥5 cm H_2_O.
- Severe ARDS: PaO_2_/FiO_2_ ≤100 mmHg on ventilators setting that include PEEP ≥5 cm H_2_O.

Severity of COVID-19 clinical form was defined using the ordinary scale, already used for influenza^2^ and COVID-19^3^. Severity of COVID-19 was defined using the following categories of the ordinary scale:

- score 3, not requiring supplemental oxygen;
- score 4, requiring supplemental oxygen;
- score 5, requiring high-flow nasal cannula (HFNC), non-invasive mechanical ventilation (NIV), or both;
- score 6, requiring extracorporeal membrane oxygenation, invasive mechanical ventilation (IMV), or both;
- score 7, death

The first two categories were excluded from our study as they indicate mild ambulatory disease (score 1-2).

Testosterone deficiency was defined as < 10 nmol/L as per US guidelines (Mulhall J, 2018).

**2.0 Sex hormones**

The levels of four sex hormones (estradiol, testosterone, progesterone, deydroepiandrosterone) were tested on the stored serum or plasma samples collected upon arrival or during the first two days from admission. The specimens were frozen at -80°C on the day of collection. The tests were executed at the IRCCS Sacro Cuore Don Calabria Hospital within 24 hours of unfreezing. The sex hormone levels were measured using commercial, chemiluminescent microparticle immunoassays (CMIA) kits according to manufacturer directions:

- Estradiol (ARCHITECT Estradiol, Abbott, Germany)
- Progesterone (ARCHITECT Progesterone, Abbott, Germany)
- Testosterone (ARCHITECT 2nd Generation Testosterone, Abbott, Germany)
- Deydroepiandrosterone (ARCHITECT DHEA-S, Abbott, Germany)

The normal range of sex hormones are reported in Table 1.

**Reference**

1. Force ADT. Acute respiratory distress syndrome: the Berlin definition. JAMA 2021;307:2526-33
2. Wang Y, Fan G, Salam A, et al. Comparative effectiveness of combined favipiravir and oseltamivir therapy versus oseltamivir monotherapy in critically ill patients with influenza virus infection. J Infect Dis 2019 December 11
3. Cao B, et al. A Trial of Lopinavir-Ritonavir in Adults Hospitalized with Severe Covid-19. N Engl J Med. 2020.
4. Mulhall J. Evaluation and management of testosterone deficiency. AUA Guidelines American

**3.0 Supplementary Table 1.** Individual comorbidities, time from onset to admission, symptoms and vital signs at baseline of patients aged 50 years and over stratified by sex (if data are known on a subset of patients, the number is indicated in parenthesis after the variable name).

| **Patients characteristics at baseline**  **n (%)** | **Overall**  **n = 120** | **Sex** | | **p-value** |
| --- | --- | --- | --- | --- |
|  |  | **Female**  **n = 52** | **Male**  **n = 68** |  |
| **Individual Comorbidity:** | **n (%)** |  |  |  |
| Hypertension | 71 (59.2) | 29 (55.8) | 42 (61.8) | 0.5079 |
| Chronic cardiac disease | 44 (36.7) | 20 (38.5) | 24 (35.3) | 0.7212 |
| Diabetes | 32 (26.7) | 10 (19.2) | 22 (32.4) | 0.1072 |
| Chronic pulmonary disease | 20 (16.7) | 5 (9.6) | 15 (22.1) | 0.086 |
| Obesity (98/120) | 22 (22.4) | 9 (22.0) | 13 (22.8) | 0.9202 |
| Chronic neurologic disorder | 18 (15.0) | 11 (21.2) | 7 (10.3) | 0.0988 |
| Malignancy | 17 (14.2) | 7 (13.5) | 10 (14.7) | 0.8464 |
| Dementia | 13 (10.8) | 8 (15.4) | 5 (7.4) | 0.2358 |
| Rheumatologic disorder | 10 (8.3) | 7 (13.5) | 3 (4.4) | 0.0994 |
| Chronic kidney disease | 10 (8.3) | 3 (5.8) | 7 (10.3) | 0.5113 |
| Mild Liver disease | 6 (5.0) | 0 (0.0) | 6 (8.8) | 0.0355 |
| Chronic hematologic disease | 5 (4.2) | 2 (3.9) | 3 (4.4) | 1 |
| Asthma | 4 (3.3) | 1 (1.9) | 3 (4.4) | 0.6323 |
| Malnutrition | 3 (2.5) | 3 (5.8) | 0 (0.0) | 0.0787 |
| Moderated Liver disease | 1 (0.8) | 0 (0.0) | 1 (1.5) | 1 |
| **Time from onset to admission, days, median (IQR)** | 5 (1-9) | 4 (1-7.5) | 6 (2-10) | 0.3509 |
| **Any Symptoms** |  |  |  |  |
| No | 17 (14.2) | 9 (17.3) | 8 (11.8) | 0.3882 |
| Yes | 103 (85.8) | 43 (82.7) | 60 (88.2) |  |
| **Individual Symptom** |  |  |  |  |
| Fever | 88 (73.3) | 35 (67.3) | 53 (77.9) | 0.1918 |
| Cough | 51 (42.5) | 19 (36.5) | 32 (47.1) | 0.2480 |
| Dyspnoea | 34 (28.3) | 13 (25) | 21 (30.9) | 0.4786 |
| Fatigue/Malaise | 25 (20.8) | 14 (26.9) | 11 (16.2) | 0.1509 |
| Diarrhoea | 15 (12.5) | 7 (13.5) | 8 (11.8) | 0.7806 |
| Unable to walk | 10 (8.3) | 4 (7.7) | 6 (8.8) | 1 |
| Myalgia | 9 (7.5) | 5 (9.6) | 4 (5.9) | 0.4988 |
| Confusion | 9 (7.5) | 1 (1.9) | 8 (11.8) | 0.0759 |
| Chest pain | 9 (7.5) | 3 (5.8) | 6 (8.8) | 0.7301 |
| Nausea/vomiting | 7 (5.8) | 4 (7.7) | 3 (4.4) | 0.4651 |
| Arthralgia | 6 (5.0) | 3 (5.8) | 3 (4.4) | 1 |
| Abdominal pain | 5 (4.2) | 3 (5.8) | 2 (2.9) | 0.6513 |
| Sore throat | 2 (1.7) | 2 (3.9) | 0 (0.0) | 0.1857 |
| Anosmia | 2 (1.7) | 1 (1.9) | 1 (1.5) | 1 |
| Headache | 2 (1.7) | 1 (1.9) | 1 (1.5) | 1 |
| Ageusia | 2 (1.7) | 1 (1.9) | 1 (1.5) | 1 |
| Rhinorrhoea | 2 (1.7) | 1 (1.9) | 1 (1.5) | 1 |
| Conjunctivitis | 1 (0.8) | 1 (1.9) | 0 (0.0) | 0.4333 |
| Skin rash | 1 (0.8) | 0 (0.0) | 1 (1.5) | 1 |
| **Vital signs, median (IQR)** |  |  |  |  |
| Temperature (118/120) | 36.8 (36.1-37.6) | 36.7 (36-37.4) | 37 (36.4-37.7) | 0.0672 |
| HR (118/120) | 85 (76.3-93.8) | 87 (80-94.5) | 84 (75.5-93) | 0.3510 |
| RR (78/120) | 20 (16.5-24.8) | 20 (18-24) | 21 (16-26.5) | 0.7219 |
| Systolic Blood Pressure (117/120) | 130 (120-145) | 132 (120-144) | 130 (120-145) | 0.7069 |
| Diastolic Blood Pressure (117/120) | 80 (65-87) | 70 (65-89.5) | 80 (69-85) | 0.5099 |
| Oxygen saturation (85/120) | 94 (92-96) | 95 (92.3-97.0) | 93.2 (91-95) | 0.0149 |

**4.0 Supplementary Table 2.** Laboratory data at baseline of patients 50 years and over stratified by sex.

| **Blood test: median (IQR)** | **Normal range value** | **Overall**  **n = 120** | **Sex** | | **p-value** |  |
| --- | --- | --- | --- | --- | --- | --- |
|  |  |  | **Male**  **n = 68** | **Female**  **n = 52** |  | **Adjusted p-value**  **(FDR)** |
| White cell count, x 10^9^/L (105/120) | 5.2-12.4 | 6.3 (4.6, 9.0) | 6.7 (4.8, 9.5) | 6.28 (4.57, 8.5) | 0.6553 | 0.7536 |
| Lymphocytes, x 10^9^/L (105/120) | 0.9-5.2 | 1.1 (0.6, 1.34) | 0.8 (0.54, 1.2) | 1.3 (0.8, 1.5) | 0.0002 | 0.0017 |
| Neutrophils, x 10^9^/L (105/120) | 1.9-8 | 4.8 (3.1, 7.3) | 5.1 (3.3, 7.6) | 4.3 (3.0, 6.6) | 0.2186 | 0.3353 |
| Haemoglobin, g/L (105/120) | 120-160 | 128 (112, 141) | 129 (113.8, 144.5) | 127 (109, 135) | 0.1846 | 0.3033 |
| Haematocrit, % (105/120) | 37-47 | 38.0 (33.8, 42.0) | 38.0 (33.7, 42.3) | 38.0 (34.0, 40.5) | 0.4501 | 0.54449 |
| Platelet count, x 10^9^/L (105/120) | 130-400 | 201 (139, 266) | 170.5 (130.3, 230.8) | 241 (171, 287) | 0.0045 | 0.0208 |
| AST, U/L (100/120) | 12-38 | 37 (23, 55) | 42 (29, 65.5) | 30 (19, 42) | 0.0099 | 0.0263 |
| ALT, U/L (99/120) | 10-44 | 27.0 (17.5, 44.5) | 33 (20, 53.5) | 22 (16, 33) | 0.0103 | 0.0263 |
| Total bilirubin, micromol/L (99/120) | 5-17 | 10.3 (8.6, 15.6) | 13.0 (9.45, 17) | 9.4 (6.5, 13.6) | 0.0037 | 0.0208 |
| Glucose, mmol/L (93/120) | 3.33-5.6 | 5.9 (4.9, 7.2) | 6.2 (5.1, 7.2) | 5.4 (4.5, 7.0) | 0.1611 | 0.2850 |
| Sodium, mmol/L (104/120) | 135-145 | 139 (135, 141) | 139 (135, 141) | 139 (135, 142) | 0.8572 | 0.8962 |
| Potassium, mmol/L (104/120) | 3.2-5.3 | 3.8 (3.6, 4.2) | 3.8 (3.6, 4.2) | 3.8 (3.5, 4.1) | 0.2804 | 0.3882 |
| Creatinine, micromol/L (104/120) | 40-90 | 79.6 (63.8, 118.0) | 94.0 (74.0, 131.4) | 69.5 (57.3, 82.5) | <0.0001 | <0.0001 |
| Urea, mmol/L (93/120) | 2.9-8.2 | 6.5 (4.7, 10.6) | 7.5 (5.2, 12.1) | 5.5 (4.5, 9.2) | 0.0278 | 0.0581 |
| C-reactive protein, mg/L (97/120) | 0-5 | 65.9 (23.1, 138.8) | 94.8 (24.1, 166.0) | 61.6 (17.0, 117.3) | 0.1490 | 0.2850 |
| LDH, U/L (90/120) | 140-250 | 270 (198.5, 358.3) | 310.0 (211.5, 447.5) | 245.0 (194.3, 308.3) | 0.0218 | 0.0501 |
| Arterial lactate, mmol/L (81/120) | 0.5-1.6 | 1.3 (1.0, 1.7) | 1.3 (1.1, 1.7) | 1.2 (1, 1.6) | 0.3500 | 0.4472 |
| CPK, U/L (80/120) | 34-145 | 116.0 (47.3, 181.5) | 154 (80, 273) | 59.0 (33.5, 126.5) | 0.0005 | 0.0037 |
| D-Dimer (73/120) | 0-500 | 782 (440, 1848) | 760 (439, 1687.5 | 2.75880 (440, 1563) | 0.9780 | 0.9780 |
| Ferritin, microg/L (69/120) | 11-306.8 | 467.8 (214.2, 872.6) | 640.6 (296, 1045) | 335.5 (108, 562) | 0.0079 | 0.0263 |
| Procalcitonin, microg/L (69/120) | 0-0.5 | 0.16 (0.1, 0.5) | 0.2 (0.1, 0.67) | 0.1 (0.1, 0.4) | 0.2869 | 0.3882 |
| IL-6, pg/mL (61/120) | 0-6.4 | 44.7 (21.2, 72.7) | 59.8 (32.9, 88.3) | 26.9 (18.3, 55.6) | 0.0086 | 0.0263 |
